# Rapidly Changing Serous Detachment During BRAF and MEK Inhibitor Therapy

**DOI:** 10.7759/cureus.20491

**Published:** 2021-12-17

**Authors:** Chisato Agata, Kohdai Kitamoto, Kohei Ueda, Keiko Azuma, Tatsuya Inoue, Ryo Obata

**Affiliations:** 1 Department of Ophthalmology, University of Tokyo Graduate School of Medicine, Tokyo, JPN; 2 Department of Ophthalmology, Yokohama City University Medical Center, Kanagawa, JPN

**Keywords:** metastatic melanoma, mek inhibitor, encorafenib, braf inhibitor, binimetinib

## Abstract

We report a case of rapidly changing serous retinal detachment (SRD) during melanoma therapy with a combination of encorafenib, a serine/threonine-protein kinase B-Raf (BRAF) inhibitor, and binimetinib, a mitogen-activated protein kinase (MEK) inhibitor. A 50-year-old woman with metastatic melanoma presented with a sudden visual blur. She had been treated with encorafenib (450 mg every morning) and binimetinib (45 mg every 12 hours) after surgery for four months. Ophthalmological examination revealed bilateral SRD, but it was completely resolved after two hours. Visual acuity was normal in each eye. Encorafenib and binimetinib were continued. Shallow SRD appeared again five months later, but it resolved in two months. MEKAR typically occurs shortly after the start of an administration, and its development after several months was very little known. Continued examination for ophthalmic events should be considered.

## Introduction

Melanoma is a most serious skin cancer because of its high tendency to spread and it arises from melanocytes, which produce pigment in the skin. The number of new cases in Japan is one to two per 100,000 [1]. Current treatment options are divided into local, regional and systemic therapy. Local therapy options include intralesional injections, topical medications and radiotherapy ablation. Regional therapy includes isolated limb perfusion and infusion. However, for stage III malignant melanoma adjuvant one year systemic therapy is standard of care, new molecularly targeted drugs such as serine/threonine-protein kinase B-Raf (BRAF) inhibitors and mitogen-activated protein kinase (MEK) inhibitors have been used in recent years. BRAF is one of the kinase proteins that constitute the mitogen-activated protein kinase (MAPK) pathway. BRAF and MAPK activate extracellular signal-regulated kinase (ERK) downstream of the MAPK pathway and regulate cell proliferation [2].

The combination therapy of encorafenib, a BRAF inhibitor, and binimetinib, a MEK inhibitor, was approved in 2018 for unresectable or metastatic malignant melanoma positive for BRAF V600E or V600K gene mutation. In an international joint phase III study (CMEK 162B2301 study Part 1), it was reported that 78 patients (40.6%) in the encorafenib/binimetinib combination group had adverse events (AEs) of eye disorders including serous retinal detachment (SRD) [3]. This AE, called as MEK-associated retinopathy (MEKAR), typically occurs shortly after the start of administration [3], and its development after several months was very little known. We herein reported a case that presented with rapidly changing SRD more than four months after the initiation of encorafenib/binimetinib therapy.

## Case presentation

A 50-year-old woman was referred to our clinic on July 29, 2020 with a chief complaint of blurred vision. Since childhood, she had chloasma on the entire surface of her right thigh. From the summer of 2018, she found an increasing mass at the same site and was referred to our dermatologist. In March 2019, she was diagnosed with malignant melanoma and underwent right femoral malignancy A resection, right inguinal sentinel lymph node biopsy, and full-thickness skin grafting. The pathological diagnosis was malignant melanoma, the size was about 30x25 mm. The sentinel node was positive for metastasis melanoma. The surgical margin was negative for melanoma. The stage was T4bN1aM0, stage IIIc, with positive BRAF v600 mutation. Pembrolizumab was administered postoperatively for 11 months but could not prevent metastasis. No delays or dose adjustments. After the first Pembrolizumab dose, she got thyrotoxicosis. CT showed multiple nodular lesions under the skin of the right thigh, which were larger than before. No brain metastases were found on MRI. Then she started to receive encorafenib 90 mg and binimetinib 450 mg on March 11, 2020. Ophthalmologic examination was unremarkable at that time. Four months later, on July 20, she complained of blurred vision and was referred to our clinic on the 29th.

**Figure 1:**
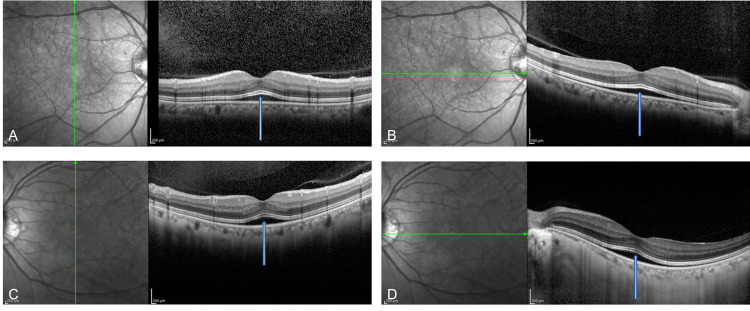
OCT obtained at around 12 am OCT images of the right (A, B) and the left (C, D) eyes were obtained at around 12 am. A symmetrical tent-like shallow SRD centered on the fovea (blue arrows) was seen in both the vertical (A, C) and the horizontal (B, C) scans. The interdigitation zone above the SRD was thickened. OCT - optical coherence tomography, SRD - serous retinal detachment

She had no specific family history. She had taken encorafenib and binimetinib at 8:30 am on that day. Her best-corrected visual acuity was 20/16 OD and 20/16 OS. Pupils, intraocular pressure, and anterior segment examinations were normal. Fundus examination demonstrated normal optic nerves, vessels, and periphery. Optical coherence tomography (OCT) obtained at around 12 am showed a tent-like shallow SRD on the fovea in both eyes. The interdigitation zone was thickened (Figures 1A-1D).

**Figure 2:**
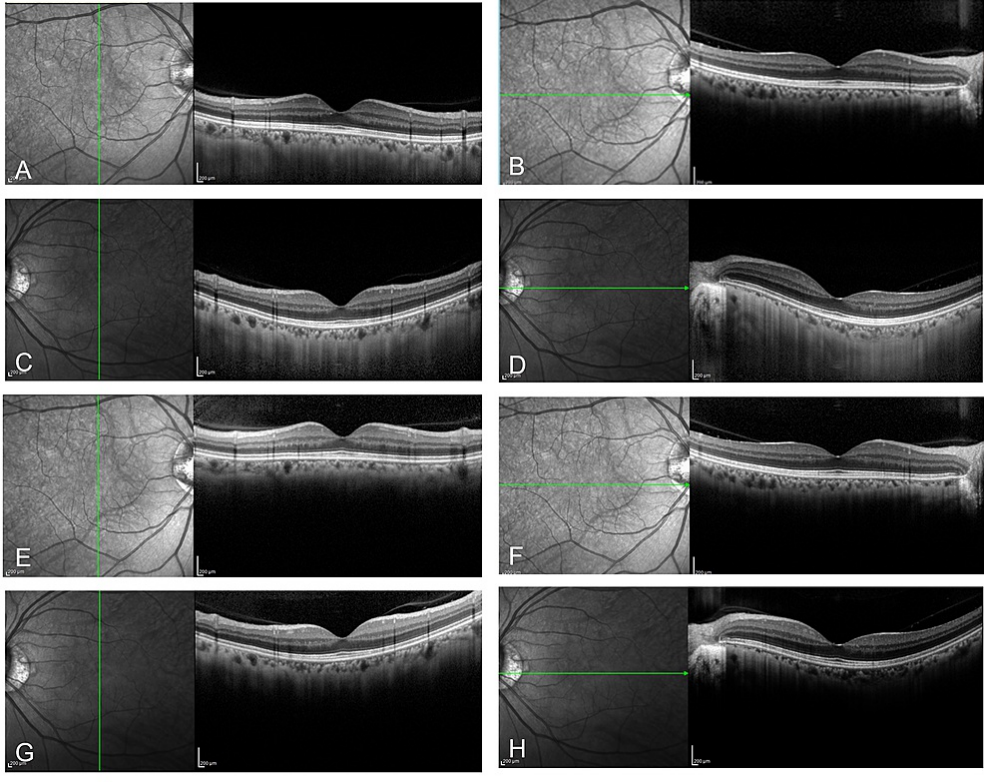
OCT re-examined after one and two hours OCT was re-examined one hour (A and B for the right eye or C and D for the left eye) and two hours (E and F for the right eye or G and H for the left eye) after the examination of the images shown in Figure 1. SRD and the thickening of the interdigitation zone were not found. OCT - optical coherence tomography, SRD - serous retinal detachment

OCT re-examined after one and two hours, but SRD and the thickening of the interdigitation zone disappeared (Figures 2A-2H).

BRAF/MEK targeted therapy was continued with careful follow-up. In August, shallow SRD was found again, but it disappeared the next month. In December, shallow SRD was observed again which disappeared next February. Humphrey 30-2 visual field tests performed in August 2020 and April showed no decrease in sensitivity in both eyes.

We measured the choroidal thickness using OCT images. The choroidal thickness when SRD was observed at the first visit was 152 μm and 129 μm in the right and left eye, respectively. It was 145 μm and 134 μm two hours after on the same day when SRD was resolved. The choroidal thickness at the final visit was 142 μm and 153 μm.

## Discussion

We report a case of rapidly changing SRD that appeared four months after starting melanoma therapy with a combination of encorafenib and binimetinib. Because no visual loss or visual field detects were observed, the therapy was continued.

MEKAR appears in more than 90% of cases treated with MEK inhibitors, and it is particularly frequent with combination therapy [3]. The onset is generally within several weeks immediately after the start of therapy, and after three to six months, it tends to improve even if chemotherapy is continued [3]. Other reports suggest a median onset is day 38 [4]. The reason why the onset time of MEKAR in the present case was atypical is unclear. It is reported that the risk of MEKAR includes old age, low glomerular filtration rate, and a history of eye diseases [5], but this case did not have such risks.

MEKAR is said to be fully reversible and the visual acuity is good even with continued drug exposure. However, distinguishing from other diseases is important [6]. The relatively late onset of MEKAR in the present case suggested that careful follow-up examination with appropriate assessment should be continued.

In this case, the SRD disappeared in about one hour. In a study of 62 cases treated with MEK inhibitors, it was reported that the average retinal thickness measured by OCT increased transiently and returned to the original value within about four hours [7]. In addition, Li et al. reported a case in which mild SRD was observed six days after encorafenib and binimetinib combination therapy, and SRD increased after four hours [8]. Such fluctuations are rare in other retinal diseases and can be said one of the characteristics of MEKAR. In the present case, SRD disappeared after one and two hours. A relatively shorter half-life of binimetinib in the blood (eight hours) than other MEK inhibitors might be an associated factor with such rapid fluctuation. Of note, the event occurred four months after starting therapy in the case, thus the fluctuation of SRD within hours should be considered when assessing MEKAR even several months after starting chemotherapy.

The mechanism of MEKAR development is still unclear. The previous report points out an increase in SRD is associated with patient anxiety and is similar to the mechanism of central serous chorioretinopathy (CSC) that can be induced by stress [8]. In the present case, it is possible that the patient had stress before presenting SRD. However, in recent years, differences were pointed out between MEKAR and CSC [5]. MEKAR occurs simultaneously and almost equally in both eyes. The SRD in OCT is not only dome-like, which is characteristic of CSC, but also caterpillar, wavy, splitting, etc. Ellipsoid zone (EZ) and interdigitation zone (IZ) are more clarified in MEKAR than CSC, retinal pigment epithelium (RPE) uplift observed in CSC are not observed in MEKAR, choroid thickness is normal and does not increase or decrease depending on the SRD, and no leakage point is observed by fluorescein angiography. Thus some other factors than patient anxiety or mental stress may be involved in the mechanism of the development of MEKAR.

## Conclusions

In conclusion, MEKAR was observed about four months after initiation of chemotherapy. It has been reported that MEKAR does not cause serious damage, and no visual loss has occurred in this case either. However, the longer-term prognosis is not well understood. MEKAR typically occurs shortly after the start of an administration, and its development after several months was very little known. In this case as well, careful follow-up is important because it cannot be ruled out that unexpected retinal abnormalities may appear over a longer period of time.
